# Racial and Ethnic Differences in 30-Day Hospital Readmissions Among US Adults With Diabetes

**DOI:** 10.1001/jamanetworkopen.2019.13249

**Published:** 2019-10-11

**Authors:** Rene Rodriguez-Gutierrez, Jeph Herrin, Kasia J. Lipska, Victor M. Montori, Nilay D. Shah, Rozalina G. McCoy

**Affiliations:** 1 Division of Endocrinology, Hospital Universitario Dr José E. Gonzalez, Universidad Autónoma de Nuevo León, Monterrey, Nuevo León, Mexico; 2 Knowledge and Evaluation Research Unit in Endocrinology, Mayo Clinic, Rochester, Minnesota; 3 Plataforma INVEST Medicina Universidad Autónoma de Nuevo León–Knowledge and Evaluation Research Unit Mayo Clinic, Universidad Autónoma de Nuevo León, Monterrey, Nuevo León, Mexico; 4 Section of Cardiovascular Medicine, Department of Internal Medicine, Yale School of Medicine, New Haven, Connecticut; 5 Flying Buttress Associates, Charlottesville, Virginia; 6 Division of Endocrinology, Department of Internal Medicine, Yale School of Medicine, New Haven, Connecticut; 7 Division of Endocrinology, Diabetes, Metabolism, and Nutrition, Department of Medicine, Mayo Clinic, Rochester, Minnesota; 8 OptumLabs, Cambridge, Massachusetts; 9 Division of Health Care Policy and Research, Department of Health Sciences Research, Mayo Clinic, Rochester, Minnesota; 10 Robert D. and Patricia E. Kern Center for the Science of Health Care Delivery, Mayo Clinic, Rochester, Minnesota; 11 Division of Community Internal Medicine Department of Medicine, Mayo Clinic, Rochester, Minnesota

## Abstract

**Importance:**

Differences in readmission rates among racial and ethnic minorities have been reported, but data among people with diabetes are lacking despite the high burden of diabetes and its complications in these populations.

**Objectives:**

To examine racial/ethnic differences in all-cause readmission among US adults with diabetes and categorize patient- and system-level factors associated with these differences.

**Design, Setting, and Participants:**

This retrospective cohort study includes 272 758 adult patients with diabetes, discharged alive from the hospital between January 1, 2009, and December 31, 2014, and stratified by race/ethnicity. An administrative claims data set of commercially insured and Medicare Advantage beneficiaries across the United States was used. Data analysis took place between October 2016 and February 2019.

**Main Outcomes and Measures:**

Unplanned all-cause readmission within 30 days of discharge and individual-, clinical-, economic-, index hospitalization–, and hospital-level risk factors for readmission.

**Results:**

A total of 467 324 index hospitalizations among 272 758 adults with diabetes (mean [SD] age, 67.7 [12.7]; 143 498 [52.6%] women) were examined. The rates of 30-day all-cause readmission were 10.2% (33 683 of 329 264) among white individuals, 12.2% (11 014 of 89 989) among black individuals, 10.9% (4151 of 38 137) among Hispanic individuals, and 9.9% (980 of 9934) among Asian individuals (*P* < .001). After adjustment for all factors, only black patients had a higher risk of readmission compared with white patients (odds ratio, 1.05; 95% CI, 1.02-1.08). This increased readmission risk among black patients was sequentially attenuated, but not entirely explained, by other demographic factors, comorbidities, income, reason for index hospitalization, or place of hospitalization. Compared with white patients, both black and Hispanic patients had the highest observed-to-expected (OE) readmission rate ratio when their income was low (annual household income <$40 000 among black patients: OE ratio, 1.11; 95% CI, 1.09-1.14; among Hispanic patients: OE ratio, 1.11; 95% CI, 1.07-1.16) and when they were hospitalized in nonprofit hospitals (black patients: OE ratio, 1.10; 95% CI, 1.08-1.12; among Hispanic patients: OE ratio, 1.08; 95% CI, 1.05-1.12), academic hospitals (black patients: OE ratio, 1.16; 95% CI, 1.13-1.20; Hispanic patients: OE ratio, 1.12; 95% CI, 1.06-1.19), or large hospitals (ie, with ≥400 beds; black patients: OE ratio, 1.11; 95% CI, 1.09-1.14; Hispanic patients: OE ratio, 1.09; 95% CI, 1.04-1.14).

**Conclusions and Relevance:**

In this study, black patients with diabetes had a significantly higher risk of readmission than members of other racial/ethnic groups. This increased risk was most pronounced among lower-income patients hospitalized in nonprofit, academic, or large hospitals. These findings reinforce the importance of identifying and addressing the many reasons for persistent racial/ethnic differences in health care quality and outcomes.

## Key Points

**Question:**

Are there differences in hospital readmission rates among racial and ethnic minorities with diabetes, and if so, what are the individual-level and hospital-level factors associated with these differences?

**Findings:**

In this cohort study of 272 758 adults with diabetes, black patients had a significantly higher risk of unplanned all-cause 30-day hospital readmission than members of other racial/ethnic groups. This difference was most pronounced among lower-income patients and patients hospitalized in nonprofit, academic, or large hospitals.

**Meaning:**

The observed differences in unplanned hospital readmission rates between black patients with diabetes and members of all other racial/ethnic groups reinforce the importance of identifying and addressing gaps in care that may be contributing to racial/ethnic disparities in health care quality and outcomes.

## Teaser

This cohort study examines racial/ethnic differences in all-cause readmission among US adults with diabetes and categorizes patient- and system-level factors associated with these differences.

## Introduction

Diabetes is among the most prevalent and costly chronic diseases in the United States, costing $327 billion annually and accounting for 1 of every 7 dollars spent on health care in the United States.^1^ Diabetes disproportionately affects racial/ethnic minorities, who also have worse diabetes-related health outcomes (ie, microvascular and macrovascular outcomes) and mortality.^2,3^ Hospitalizations contribute substantially to the cost and burden of diabetes care, as people with diabetes are more likely to be hospitalized and experience unplanned readmissions than people without diabetes.^4,5,6,7,8^ Prior studies have identified persistent disparities in readmission risk among black and Hispanic adults with diabetes, which seemed to correlate with underlying social determinants of health.^5,6,9,10,11^ Nevertheless, none of these studies assessed the association with race separately from other important risk factors for readmission, such as age, comorbidity, economic status, reasons for hospitalization, and site of care. Hence, our understanding of individual and hospital factors contributing to these disparities remains uncertain and is an essential first step in developing policy and practice interventions aimed at improving health outcomes for all people with diabetes.

There are several, likely interrelated, reasons why members of a racial/ethnic minority may have higher rates of unplanned hospital readmission, and it has been difficult to differentiate among them.^5,6,12^ Specifically, readmission risk can be increased by person-level factors, such as age, sex, and comorbidity burden.^13^ Hospitalization-related factors, specifically the reason for index admission (ie, initial or first admission), length of stay, or history of recurrent hospitalizations, can increase readmission risk.^14^ Socioeconomic factors, including income, education level, insurance status, and availability of a social support network and community resources, also play a role.^14,15^ Finally, factors related to the quality of care received at the discharging hospital (ie, the site where the person received their care) can affect subsequent readmission risk.^16^ Improving care quality by reducing readmissions requires a nuanced understanding of the racial/ethnic disparities that may drive differences in readmission risk, including the interplay of these contributing variables.

Using a large data set of commercially insured and Medicare Advantage beneficiaries across the United States, we examined the rates of unplanned 30-day hospital readmissions among adults with diabetes, focusing specifically on 4 ethnic/racial groups derived from US Census categories: white individuals, black individuals, Hispanic individuals, and Asian individuals. We further evaluated factors associated with readmissions across these different groups, focusing specifically on the 5 following prespecified categories: demographic characteristics, clinical factors, economic status, aspects of the index hospitalization, and characteristics of the hospital where the index hospitalization took place. Using a nationwide administrative claims database for this work allowed us to address these questions in ways not possible with publicly available hospital and US Census data, including isolating within-hospital differences and accounting for the patient-level clinical and socioeconomic factors that are the focus of this study.

## Methods

### Data Source

We conducted retrospective analysis of data from the OptumLabsData Warehouse, an administrative claims database of private and Medicare Advantage enrollees across the United States, with the greatest representation in the South.^17,18^ Study data were accessed using techniques compliant with the Health Insurance Portability and Accountability Act of 1996, and because this study involved analysis of preexisting, deidentified data, it was exempt from institutional review board approval. Patient consent was not obtained because all presented data were anonymized prior to data set creation. This retrospective cohort study was performed following the Strengthening the Reporting of Observational Studies in Epidemiology (STROBE) reporting guideline.

### Study Participants

We included adults with an established diagnosis of diabetes who experienced an index hospitalization (ie, first-time hospital admission not preceded by another hospitalization within a 30-day period) for any cause between January 1, 2009, and December 31, 2014, were discharged alive, and had at least 12 months of enrollment in private insurance or Medicare Advantage prior to index admission and at least 31 days after the index discharge date (ie, date discharged from hospital after the index admission). Hospitalizations for the principal discharge diagnoses of medical treatment of cancer, psychiatric disease, and pregnancy were excluded in accordance with hospitalwide readmission measure methods.^19,20^ Principal diagnoses for index hospitalizations were identified using the primary diagnosis *International Classification of Diseases, Ninth Revision, Clinical Modification *(*ICD-9-CM*) codes from the hospitalization claims and grouped using the Agency for Healthcare Research and Quality Comorbidity and Clinical Classifications Software.^21^ We excluded admissions with lengths of stay longer than 365 days.

Diagnosis of diabetes was established by applying National Committee for Quality Assurance Healthcare Effectiveness Data and Information Set claims–computable criteria^22^ to data from the 12 months preceding the index hospitalization; patients with only gestational diabetes (*ICD-9-CM* codes, 648.0 and 648.8) were not included. Race/ethnicity was ascertained from OptumLabsData Warehouse enrollment files, and individuals for whom race/ethnicity was missing or unknown (10 970 of 283 729 patients [3.9%]) were excluded.

### Independent Variables

Risk factors for readmission for each racial/ethnic group were categorized as being primarily associated with demographic factors, clinical factors, economic factors, the index hospitalization, or the index hospital. Demographic factors included age at the index hospitalization and sex, both obtained from enrollment data. Clinical factors included the component conditions of the Diabetes Complications Severity Index^23,24^ and the Charlson Comorbidity Index (ie, dementia; chronic pulmonary disease; rheumatologic disease; peptic ulcer disease; mild liver disease; moderate or severe liver disease; hemiplegia or paraplegia; cancer; metastatic carcinoma; and HIV/AIDS),^25^ all of which were calculated using diagnoses during the 12 months before the index admission, the Diabetes Complication Severity Index itself, and treatment with insulin, which was ascertained from pharmacy claims within 120 days of the index admission. Annual household income was used a marker of economic factors. Factors related to the index hospitalization included length of stay (ie, 1, 2-4, 5-7, 8-14, and ≥15 days), discharge year, and history of another hospitalization during the prior 12 months. Factors related to the hospital were obtained from the American Hospital Association Survey data file^26^ and included hospital ownership status (ie, public, nonprofit, or for profit), teaching status (ie, nonteaching, residency-only, or academic medical center), urban status (ie, division, in or adjacent to metropolitan area with ≥2.5 million people; metropolitan, area with ≥50 000 to 2.5 million people; micropolitan, area with ≥10,000 to <50 000 people; and rural, area with <10 000 people); sole community hospital status; and size (ie, <50, 50-199, 200-399, and ≥400 beds).

### Primary Outcome

Our primary outcome was unplanned readmission for any cause within 30 days of discharge. Readmissions were identified using inpatient claims in the OptumLabsData Warehouse; we excluded readmissions that were classified as planned by the Centers for Medicare & Medicaid Services planned readmission algorithm.^19,20^ We also excluded readmissions within 1 day of index admission discharge, as these may have represented continuation of the index admission.

### Statistical Analysis

We summarized patient and admission characteristics overall and by race/ethnicity for all index admissions and readmissions, testing for differences in characteristics using χ^2^ tests of independence (null hypothesis, no difference among racial/ethnic groups). For each racial/ethnic group, we identified the 10 most common causes for admission and readmission, reporting the number and percentage for each.

We then assessed the association of patient risk factors with readmission risk using mixed-effects logistic regression models. To assess the relative association of clinical, admission, economic, and hospital factors to differences in risk, we estimated 5 nested models. Starting with an empty model that included only indicators for race/ethnicity, patient demographic characteristics (ie, age and sex), calendar year, and random effect for hospital (model 0), we added 4 models, sequentially: (1) clinical factors, comprising individual comorbidities and insulin use; (2) index hospitalization factors, comprising length of stay, prior hospitalization, planned admission status, and discharge year; (3) economic factors, comprising annual household income; and (4) hospital factors, comprising hospital ownership status, teaching status, urban category status, sole community hospital status, and bed number. For each model, we reported the readmission odds ratio (OR) for each racial/ethnic group. By examining how the association of race with readmission changes across models, we could evaluate the importance of each set of factors in explaining racial differences.

Finally, to estimate within-hospital disparity effects, we used the Peters-Belsen approach, which was developed for understanding wage disparities and has been used previously to assess health care disparities.^27,28^ Specifically, we estimated a logistic regression model corresponding to model 2 (without calendar year) using only white patients and used this model to estimate an expected probability of readmission for all patients in the cohort. For each hospital, we used these expected probabilities to calculate the observed-to-expected (OE) ratio of readmission for each racial/ethnic group at each hospital. We used model 2 rather than model 4 so that we could evaluate differences in the OE ratio across subgroups of patients according to income, hospital characteristics, and calendar year. After using the model to calculate an expected readmission risk for all patients, the observed and expected values were summed over the subgroups of interest to calculate OE ratio for that group. These were reported with 95% CIs based on the delta method for income category, year, and hospital characteristics.

We used multiple imputation with 20 imputations to account for missing income data (the only variable with missing values; missing in 18 498 of 272 758 patients [6.8%] or 31 819 of 467 324 [6.8%] of admissions) in all models. We imputed this under the assumption that data were missing at random, using patient race/ethnicity, readmission status, and clinical and demographic factors in the imputation model. *P* < .05 was considered statistically significant, and all tests were 2-tailed. All analyses were conducted using SAS statistical software version 9.3 (SAS Institute) and Stata version 13.1 (StataCorp).

## Results

### Study Population

We identified 272 758 adults with diabetes who experienced 467 324 index hospitalizations during the 6-year study period (Table 1). Mean (SD) age was 67.7 (12.7) years overall, 69.2 (12.2) years among white patients, 67.6 (12.5) years among black patients, 67.1 (13.8) years among Hispanic patients, and 69.2 (13.6) years among Asian patients (*P* < .001). Overall, 143 498 patients (52.6%) were women. Among admissions for white patients, 169 703 (51.5%) were for women; among black patients, 54 965 (61.1%); among Hispanic patients, 20 598 (54.0%); and among Asian patients, 5107 (51.4%) (*P* < .001). Of 467 324 index hospitalizations, 329 264 (70.4%) of hospitalizations were among white patients, 89 989 (19.2%) among black patients, 38 137 (8.1%) among Hispanic patients, and 9934 (2.1%) among Asian patients. There was marked heterogeneity in nearly all characteristics of these racial/ethnic groups. Black and Hispanic patients tended to be younger and female and have lower annual household incomes than white or Asian patients (annual household income <$40 000: black patients, 50 590 [56.2%]; Hispanic patients, 16 852 [44.2%]; white patients, 131 071 [39.8%]; Asian patients 2895 [29.1%]; *P* < .001). Black patients had a greater prevalence of diabetes-related complications (Diabetes Complications Severity Index ≥7: among black patients, 17 489 [19.4%]; among white patients, 53 489 [16.2%]; among Hispanic patients, 6038 [15.8%]; among Asian patients, 1299 [13.1%]; *P* < .001). Similarly, insulin use was highest among black and Hispanic patients (black patients, 27 104 [30.1%]; Hispanic patients, 11 200 [29.4%]; white patients, 85 533 [26.0%]; Asian patients, 2062 [20.8%]; *P* < .001). Black patients were also more likely than any other group to have been hospitalized in public hospitals (10 568 [11.7%]), academic hospitals (23 415 [26.0]%), metropolitan hospitals (72 834 [80.9%]), and large hospitals (ie, >400 beds; 43 502 [48.3%]) (*P* < .001).

**Table 1.** Baseline Characteristics of the Study Population at Index Hospital Admission

Characteristic	No. (%)^a^				*P* Value
	White Patients (n = 193 793)	Black Patients (n = 49 288)	Hispanic Patients (n = 23 206)	Asian Patients (n = 6471)	
Index admissions, No.	329 264	89 989	38 137	9934	NA
Demographic factors					
Patient age, mean (SD), y	69.2 (12.2)	67.6 (12.5)	67.1 (13.8)	69.2 (13.6)	<.001
Patient age category, y					
<45	12 844 (3.9)	4627 (5.1)	2836 (7.4)	666 (6.7)	<.001
45-64	90 878 (27.6)	27 957 (31.1)	11 252 (29.5)	2257 (22.7)	
65-74	101 251 (30.8)	27 580 (30.6)	10 611 (27.8)	2948 (29.7)	
≥75	124 291 (37.7)	29 825 (33.1)	13 438 (35.2)	4063 (40.9)	
Women	169 703 (51.5)	54 965 (61.1)	20 598 (54.0)	5107 (51.4)	<.001
Economic factors					
Annual household income, US$					
<40 000	131 071 (39.8)	50 590 (56.2)	16 852 (44.2)	2895 (29.1)	<.001
40 000-49 999	33 329 (10.1)	9268 (10.3)	3574 (9.4)	836 (8.4)	
50 000-59 999	28 575 (8.7)	7020 (7.8)	3071 (8.1)	782 (7.9)	
60 000-74 999	32 573 (9.9)	6590 (7.3)	3322 (8.7)	857 (8.6)	
75 000-99 999	36 538 (11.1)	5895 (6.6)	3475 (9.1)	1133 (11.4)	
≥100 000	46 815 (14.2)	4525 (5.0)	3665 (9.6)	2254 (22.7)	
Factors related to the index hospitalization					
Length of stay, d					
1	46 136 (14.0)	10 944 (12.2)	5253 (13.8)	1432 (14.4)	<.001
2-4	178 206 (54.1)	46 411 (51.6)	20 638 (54.1)	5052 (50.9)	
5-7	64 392 (19.6)	19 049 (21.2)	7399 (19.4)	1971 (19.8)	
8-14	32 565 (9.9)	10 578 (11.8)	3867 (10.1)	1120 (11.3)	
≥15	7965 (2.4)	3007 (3.3)	980 (2.6)	359 (3.6)	
Prior admission(s) in past year	136 394 (41.4)	41 512 (46.1)	15 397 (40.4)	3598 (36.2)	<.001
Planned admission	54 268 (16.5)	11 355 (12.6)	5801 (15.2)	1629 (16.4)	<.001
Clinical factors					
Comorbidities					
Dementia	11 867 (3.6)	4182 (4.6)	1728 (4.5)	466 (4.7)	<.001
Chronic lung disease	129 628 (39.4)	35 409 (39.3)	13 403 (35.1)	2989 (30.1)	<.001
Rheumatologic disease	15 358 (4.7)	4627 (5.1)	1934 (5.1)	338 (3.4)	<.001
Peptic ulcer disease	10 642 (3.2)	3100 (3.4)	1407 (3.7)	393 (4.0)	<.001
Mild liver disease	8021 (2.4)	1740 (1.9)	1407 (3.7)	234 (2.4)	<.001
Moderate or severe liver disease	5498 (1.7)	1089 (1.2)	941 (2.5)	145 (1.5)	<.001
Hemiplegia or paraplegia	7709 (2.3)	3062 (3.4)	989 (2.6)	348 (3.5)	<.001
Cancer	43 235 (13.1)	10 977 (12.2)	4272 (11.2)	1130 (11.4)	<.001
Metastatic carcinoma	7449 (2.3)	1993 (2.2)	821 (2.2)	221 (2.2)	.51
HIV/AIDS	527 (0.2)	552 (0.6)	145 (0.4)	11 (0.1)	<.001
Diabetes-related complications					
Retinopathy	31 717 (9.6)	10 969 (12.2)	4550 (11.9)	1053 (10.6)	<.001
Nephropathy	73 322 (22.3)	24 746 (27.5)	8768 (23.0)	2618 (26.4)	<.001
Neuropathy	106 605 (32.4)	29 138 (32.4)	11 455 (30.0)	2317 (23.3)	<.001
Heart failure	101 625 (30.9)	32 608 (36.2)	10 510 (27.6)	2722 (27.4)	<.001
Cerebrovascular disease	147 984 (44.9)	38 926 (43.3)	15 495 (40.6)	3991 (40.2)	<.001
Peripheral vascular disease	68 420 (20.8)	20 227 (22.5)	7690 (20.2)	1561 (15.7)	<.001
Metabolic complications	7370 (2.2)	3034 (3.4)	1002 (2.6)	216 (2.2)	<.001
Diabetes Complications Severity Index Category					
0	78 301 (23.8)	18 986 (21.1)	10 399 (27.3)	2820 (28.4)	<.001
1-2	85 893 (26.1)	22 196 (24.7)	9691 (25.4)	2711 (27.3)	
3-6	111 581 (33.9)	31 318 (34.8)	12 009 (31.5)	3104 (31.2)	
≥7	53 489 (16.2)	17 489 (19.4)	6038 (15.8)	1299 (13.1)	
Insulin use	85 533 (26.0)	27 104 (30.1)	11 200 (29.4)	2062 (20.8)	<.001
Factors related to the index hospital					
Ownership type					
Public	25 968 (7.9)	10 568 (11.7)	2836 (7.4)	815 (8.2)	<.001
Nonprofit	297 201 (90.3)	77 935 (86.6)	30 727 (80.6)	8992 (90.5)	
For profit	6095 (1.9)	1486 (1.7)	4574 (12.0)	127 (1.3)	
Teaching status^b^					
Nonteaching	186 854 (56.7)	39 649 (44.1)	20 656 (54.2)	4156 (41.8)	<.001
Residency	82 997 (25.2)	26 925 (29.9)	8939 (23.4)	3539 (35.6)	
Academic	59 413 (18.0)	23 415 (26.0)	8542 (22.4)	2239 (22.5)	
Urban category^c^					
Division	36 067 (11.0)	11 388 (12.7)	10 298 (27.0)	3016 (30.4)	<.001
Metropolitan	259 699 (78.9)	72 834 (80.9)	26 022 (68.2)	6290 (63.3)	
Micropolitan	25 269 (7.7)	4484 (5.0)	1561 (4.1)	560 (5.6)	
Rural	8229 (2.5)	1283 (1.4)	256 (0.7)	68 (0.7)	
Sole community hospital status					
No	318 190 (96.6)	88 511 (98.4)	36 828 (96.6)	9503 (95.7)	<.001
Yes	11 049 (3.4)	1472 (1.6)	1309 (3.4)	430 (4.3)	
Unknown	25 (0.1)	0	0	0	
Size, No. of beds					
<50	11 165 (3.4)	1534 (1.7)	709 (1.9)	135 (1.4)	<.001
50-199	90 812 (27.6)	15 100 (16.8)	9848 (25.8)	2852 (28.7)	
200-399	112 524 (34.2)	29 853 (33.2)	13 920 (36.5)	2755 (27.7)	
≥400	114 763 (34.9)	43 502 (48.3)	13 660 (35.8)	4192 (42.2)	

^a^ Counts and percentages reported for index hospitalizations.

^b^ Nonteaching indicates no resident training and the hospital does not belong to or partner with a university; residency, hospital has formal resident training programs but does not belong to a university; and academic, hospital belongs to a university and trains residents.

^c^ Division indicates in or adjacent to area with at least 2.5 million people; metropolitan, area with at least 50 000 and less than 2.5 million people; micropolitan, area with at least 10 000 and less than 50 000 people; and rural, area with less than 10 000 people.

### Reasons for Index Hospitalization

Diabetes was the most common cause for hospitalization among Hispanic patients (1952 of 38 137 admissions [5.1%]) and the second most common cause among black patients (5236 of 89 989 admissions [5.8%]), but ranked fourth among white patients (13 626 of 329 264 admissions [4.1%]) and fifth among Asian patients (358 of 9934 admissions [3.6%]) (eTable in the Supplement). The most common cause for index hospitalization among white patients was osteoarthritis (18 039 [5.5%]) and among Asian patients, sepsis (655 [6.6%]). Congestive heart failure was a common cause of hospitalization among all groups, ranking first among black patients (6109 [6.8%]), second among white patients (16 926 [5.5%]) and Hispanic patients (1856 [4.9%]), and third among Asian patients (470 [4.7%]).

### All-Cause Readmission Risk

Rates of 30-day all-cause hospital readmission were 10.2% (33 683 of 329 264) among white patients, 12.2% (11 014 of 89 989) among black patients, 10.9% (4151 of 38 137) among Hispanic patients, and 9.9% (980 of 9934) among Asian patients (*P* < .001). In the fully adjusted analysis, black patients had a higher risk of readmission compared with white patients (OR 1.05; 95% CI, 1.02-1.08) with no statistically significant increase in risk among Hispanic and Asian patients (Hispanic patients: OR, 1.02; 95% CI, 0.98-1.06; Asian patients: OR, 0.98; 95% CI, 0.91-1.05) (Table 2). The following variables were also significantly associated with readmission risk: younger age (65-74 years and ≥75 years compared with <45 years: OR, 0.90; 95% CI, 0.85-0.95), diabetes-related complications (eg, nephropathy: OR 1.23; 95% CI, 1.20-1.26; *P* < .001; heart failure: OR, 1.29; 95% CI, 1.26-1.32; *P* < .001; metabolic complications: OR, 1.21; 95% CI, 1.14-1.28; *P* < .001), all examined comorbidities with the exception of dementia and HIV/AIDS (eg, moderate or severe liver disease: OR, 1.38; 95% CI, 1.28-1.50; *P* < .001; metastatic carcinoma: OR 1.30; 95% CI, 1.23-1.38; *P* < .001), insulin use (OR, 1.07; 95% CI, 1.05-1.10; *P* < .001), prior hospitalizations (OR, 1.58; 95% CI, 1.54-1.61; *P* < .001), and lower annual income (≥$100 000 compared with <$40 000: OR, 0.90; 95% CI, 0.87-0.93; *P* < .001). Patients hospitalized in teaching hospitals compared with nonteaching hospitals (residency-only: OR, 0.96; 95% CI, 0.91-1.00; academic: OR, 1.09; 95% CI, 1.02-1.16; *P* < .001), hospitals located in larger urban areas (rural hospitals compared with division hospitals: OR, 0.83; 95% CI, 0.75-0.92; *P* = .003), and larger hospitals (≥400 beds compared with <50 beds: OR, 1.15; 95% CI, 1.04-1.26; *P* = .03) all had significantly higher odds of readmission.

**Table 2.** Risk Factors for All-cause Readmission

Factor	OR (95% CI)	*P* Value
Demographic factors		
Age category, y		
<45	1 [Reference]	NA
45-64	0.94 (0.89-0.99)	<.001
65-74	0.90 (0.85-0.95)	
≥75	0.90 (0.85-0.95)	
Sex		
Male	1 [Reference]	NA
Female	1.01 (0.99-1.03)	.48
Race/ethnicity		
White	1 [Reference]	NA
Black	1.05 (1.02-1.08)	.002
Hispanic	1.02 (0.98-1.06)	
Asian	0.98 (0.91-1.05)	
Economic factors		
Annual household income, US$		
<40 000	1 [Reference]	NA
40 000-49 999	0.96 (0.93-0.99)	<.001
50 000-59 999	1.00 (0.97-1.04)	
60 000-74 999	0.96 (0.93-0.99)	
75 000-99 999	0.96 (0.92-0.99)	
≥100 000	0.90 (0.87-0.93)	
Factors related to index hospitalization		
Length of stay, d		
2-4	1 [Reference]	NA
1	0.79 (0.76-0.81)	<.001
5-7	1.30 (1.26-1.33)	
8-14	1.66 (1.61-1.71)	
≥15	2.31 (2.20-2.42)	
Prior hospitalization(s) in past year		
0	1 [Reference]	NA
≥1	1.58 (1.54-1.61)	<.001
Planned admission		
No	1 [Reference]	NA
Yes	0.82 (0.79-0.84)	<.001
Year of discharge		
2009	1 [Reference]	NA
2010	1.01 (0.97-1.05)	.62
2011	1.01 (0.97-1.05)	.69
2012	0.99 (0.95-1.02)	.50
2013	0.99 (0.96-1.03)	.58
2014	1.00 (0.96-1.04)	.93
Clinical factors		
Comorbidities		
No comorbidities	1 [Reference]	NA
Dementia	1.02 (0.97-1.07)	.45
Chronic pulmonary disease	1.18 (1.15-1.20)	<.001
Rheumatologic disease	1.11 (1.07-1.16)	<.001
Peptic ulcer disease	1.12 (1.07-1.17)	<.001
Mild liver disease	1.31 (1.22-1.40)	<.001
Moderate or severe liver disease	1.38 (1.28-1.50)	<.001
Hemiplegia or paraplegia	1.10 (1.04-1.16)	.001
Cancer	1.11 (1.08-1.14)	<.001
Metastatic carcinoma	1.30 (1.23-1.38)	<.001
HIV/AIDS	1.16 (0.98-1.36)	.08
Diabetes-related complications		
No diabetes-related complications	1 [Reference]	NA
Retinopathy	1.03 (1.00-1.07)	.03
Nephropathy	1.23 (1.20-1.26)	<.001
Neuropathy	1.12 (1.09-1.14)	<.001
Heart failure	1.29 (1.26-1.32)	<.001
Cerebrovascular disease	1.11 (1.09-1.14)	<.001
Peripheral vascular disease	1.06 (1.03-1.08)	<.001
Metabolic complications	1.21 (1.14-1.28)	<.001
Insulin use		
No	1 [Reference]	NA
Yes	1.07 (1.05-1.10)	<.001
Factors related to the index hospital		
Hospital ownership		
Public	1 [Reference]	NA
Nonprofit	1.01 (0.95-1.06)	.18
For profit	0.92 (0.82-1.02)	
Teaching status^a^		
Nonteaching	1 [Reference]	NA
Residency only	0.96 (0.91-1.00)	<.001
Academic	1.09 (1.02-1.16)	
Urban category^b^		
Division	1 [Reference]	NA
Metro	0.97 (0.92-1.01)	.003
Micro	0.90 (0.84-0.98)	
Rural	0.83 (0.75-0.92)	
Sole community hospital status		
No	1 [Reference]	NA
Yes	0.97 (0.89-1.06)	.70
Unknown	1.39 (0.39-4.95)	
Size, No. of beds		
<50	1 [Reference]	NA
50-199	1.11 (1.02-1.20)	.03
200-399	1.13 (1.04-1.23)	
≥400	1.15 (1.04-1.26)	

Abbreviations: NA, not applicable; OR, odds ratio.

^a^ Nonteaching indicates no resident training and the hospital does not belong to or partner with a university; residency, hospital has formal resident training programs but does not belong to a university; and academic, hospital belongs to a university and trains residents.

^b^ Division indicates in or adjacent to area with at least 2.5 million people; metropolitan, area with at least 50 000 and less than 2.5 million people; micropolitan, area with at least 10 000 and less than 50 000 people; and rural, area with less than 10 000 people.

### Relative Contributions of the Readmission Risk Factor Categories

Results of the nested models are illustrated in the Figure, examining the changing association of race with readmission risk as each set of factors is sequentially added to the analysis. In the first model (model 0), only patient age, sex, and race/ethnicity (the demographic characteristics category) were considered. In this model, black patients had a higher risk of readmission compared with white patients (OR, 1.15; 95% CI, 1.12-1.18; *P* < .001), while Asian patients had a lower risk (OR, 0.89; 95% CI, 0.83-0.96; *P* < .001). The lower readmission risk among Asian patients was no longer observed when disease-level factors were added (model 1) (OR, 0.97; 95% CI, 0.90-1.05; *P* < .001), indicating that their lower risk can be explained by lower clinical complexity. The higher risk among black patients persisted (OR, 1.10; 95% CI, 1.07-1.13; *P* < .001). Model 2 incorporated data on the index hospitalization, and this only slightly attenuated the increased readmission risk among black patients (OR, 1.07; 95% CI, 1.04-1.10; *P* < .001). Subsequent incorporation of annual household income, an indicator of financial factors, in model 3 brought the risk among black patients down to an OR of 1.06 (95% CI, 1.03-1.09; *P* < .001) compared with white patients. Finally, consideration of hospital characteristics of the discharging facility (model 4) did not change the increase in readmission risk among black patients (OR 1.05; 95% CI, 1.02-1.08; *P* < .002), and their higher risk of readmission compared with white patients persisted.

**Figure.**
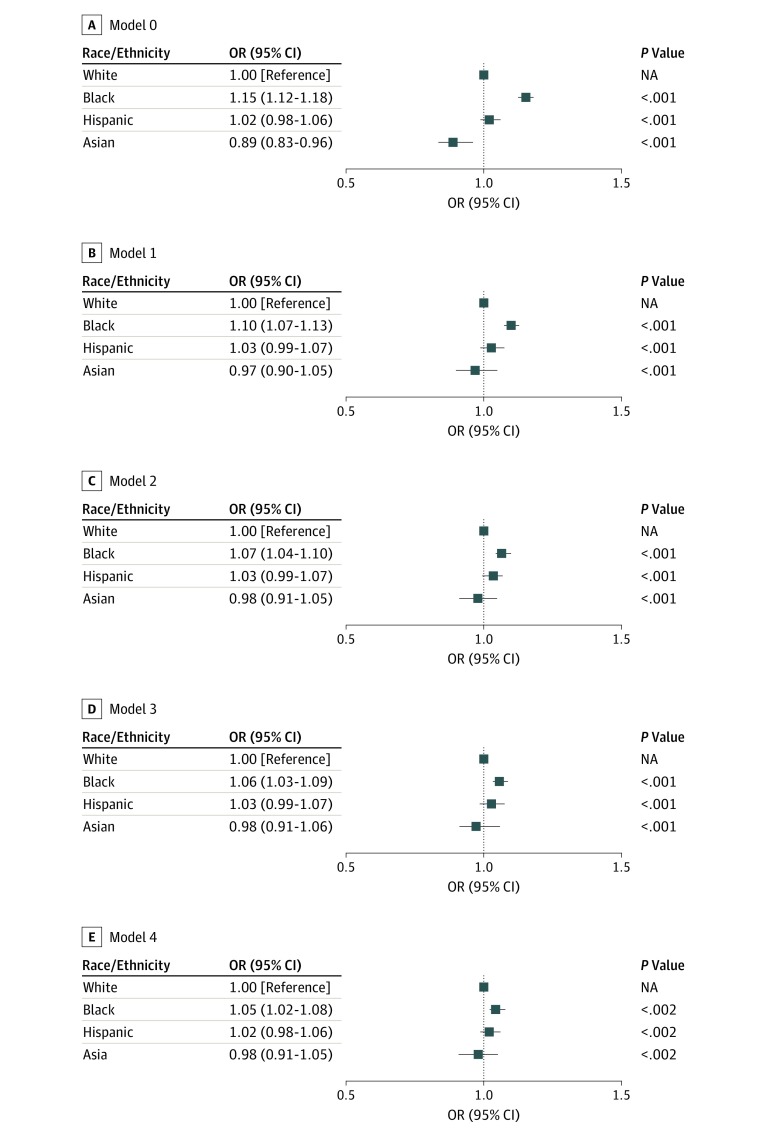
Relative Contributions of the 5 Risk Factor Categories on Observed Disparities in Readmission Risk A, Adjusted for demographic characteristics (age and sex). B, Adjusted for demographic characteristics and clinical factors (ie, individual comorbidities, complications associated with diabetes, and insulin use). C, Adjusted for demographic characteristics, clinical factors, and index hospitalization factors (ie, length of stay, discharge year, and prior hospitalization). D, Adjusted for demographic characteristics, clinical factors, index hospitalization factors, and economic factors (ie, annual household income). E, Adjusted for demographic characteristics, clinical factors, index hospitalization factors, economic factors, and hospital factors (ie, hospital ownership status, teaching status, urban status, sole community hospital status, and bed number). NA indicates not applicable; OR, odds ratio.

### Association of Financial and Hospital Factors With Readmission Risk Among Different Racial/Ethnic Groups

The OE readmission rate ratios for each racial/ethnic group are reported in Table 3. We found that lower income (ie, <$40 000) disproportionately increased readmission risk among black patients (OE ratio, 1.11; 95% CI, 1.09-1.14) and Hispanic patients (OE ratio, 1.11; 95% CI, 1.07-1.16). Black and Hispanic patients also had a disproportionately high readmission risk when hospitalized in nonprofit hospitals (black patients: OE ratio, 1.10; 95% CI, 1.08-1.12; Hispanic patients: OE ratio, 1.08; 95% CI, 1.05-1.12) and hospitals with at least 400 beds (black patients: OE ratio, 1.11; 95% CI, 1.09-1.14; Hispanic patients: OE ratio, 1.09; 95% CI, 1.04-1.14) while other groups were not affected by this. In general, black patients had a disproportionately high readmission rate no matter where they were hospitalized (eg, nonprofit: OE ratio, 1.10; 95% CI, 1.08-1.12; nonacademic: OE ratio, 1.07; 95% CI, 1.04-1.10; division: OE ratio, 1.10; 95% CI, 1.05-1.16) with the exception of rural and micropolitan hospitals, in which the OE readmission rate ratio among black patients was as expected (rural: OE ratio, 0.91; 95% CI, 0.76-1.07; micropolitan: OE ratio, 1.06; 95% CI, 0.97-1.14). All patients had a higher than expected readmission rate after index hospitalizations in academic medical centers (white patients: OE ratio, 1.07; 95% CI, 1.05 to 1.10; black patients: OE ratio, 1.16; 95% CI, 1.13-1.20; Hispanic patients: OE ratio, 1.12; 95% CI, 1.06-1.19; Asian patients, OE ratio, 1.06; 95% CI, 0.93-1.19) and hospitals with at least 400 beds (white patients: OE ratio, 1.02; 95% CI, 1.00-1.04; black patients: OE ratio, 1.11; 95% CI, 1.09-1.14; Hispanic patients: OE ratio, 1.09; 95% CI, 1.04-1.14; Asian patients: OE ratio: 1.06; 0.97-1.16).

**Table 3.** OE Readmission Rate Ratio for Each Racial/Ethnic Group in Different Economic Circumstances and Hospital Settings

Factor	OE Ratio (95% CI)			
	White Patients	Black Patients	Hispanic Patients	Asian Patients
**Economic Factors**				
Annual household income, US$				
<40 000	1.02 (1.01 to 1.04)	1.11 (1.09 to 1.14)	1.11 (1.07 to 1.16)	1.07 (0.96 to 1.18)
40 000-49 999	0.99 (0.96 to 1.02)	1.03 (0.97 to 1.09)	1.07 (0.97 to 1.17)	0.81 (0.63 to 1.00)
50 000-59 999	1.03 (0.99 to 1.06)	1.09 (1.02 to 1.16)	1.05 (0.94 to 1.16)	1.19 (0.96 to 1.42)
60 000-74 999	0.99 (0.96 to 1.02)	1.07 (0.99 to 1.14)	1.04 (0.93 to 1.14)	1.05 (0.85 to 1.25)
75 000-99 999	0.98 (0.94 to 1.01)	1.10 (1.02 to 1.18)	1.03 (0.93 to 1.13)	0.99 (0.81 to 1.17)
≥100 000	0.93 (0.91 to 0.96)	1.02 (0.93 to 1.11)	0.90 (0.80 to 1.00)	0.96 (0.84 to 1.09)
**Hospital-Level Factors**				
Ownership status				
Public	1.01 (0.97 to 1.04)	1.06 (1.01 to 1.12)	1.02 (0.91 to 1.13)	0.95 (0.75 to 1.15)
Nonprofit	1.00 (0.99 to 1.01)	1.10 (1.08 to 1.12)	1.08 (1.05 to 1.12)	1.02 (0.95 to 1.08)
For profit	0.89 (0.82 to 0.97)	1.09 (0.93 to 1.24)	0.99 (0.91 to 1.08)	0.98 (0.40 to 1.55)
Teaching status^a^				
Nonteaching	1.00 (0.98 to 1.01)	1.07 (1.04 to 1.10)	1.02 (0.98 to 1.06)	0.93 (0.84 to 1.02)
Residency only	0.95 (0.93 to 0.97)	1.06 (1.03 to 1.10)	1.11 (1.05 to 1.17)	1.07 (0.97 to 1.18)
Academic	1.07 (1.05 to 1.10)	1.16 (1.13 to 1.20)	1.12 (1.06 to 1.19)	1.06 (0.93 to 1.19)
Urban category^b^				
Division	1.05 (1.02 to 1.08)	1.10 (1.05 to 1.16)	1.03 (0.98 to 1.09)	1.00 (0.89 to 1.11)
Metropolitan	1.00 (0.99 to 1.02)	1.10 (1.08 to 1.12)	1.09 (1.05 to 1.12)	1.00 (0.93 to 1.08)
Micropolitan	0.93 (0.90 to 0.97)	1.06 (0.97 to 1.14)	1.00 (0.86 to 1.15)	1.14 (0.90 to 1.39)
Rural	0.83 (0.77 to 0.89)	0.91 (0.76 to 1.07)	0.78 (0.43 to 1.14)	0.97 (0.25 to 1.69)
Sole community hospital				
No	1.00 (0.99 to 1.01)	1.10 (1.08 to 1.11)	1.07 (1.04 to 1.10)	1.00 (0.94 to 1.06)
Yes	0.88 (0.83 to 0.93)	0.96 (0.81 to 1.10)	1.02 (0.86 to 1.18)	1.23 (0.93 to 1.52)
Unknown	0.52 (−0.52 to 1.56)	3.07 (−3.05 to 9.20)	NA	13.92 (13.92 to 13.92)
Size, No. of beds				
<50	0.85 (0.80 to 0.91)	0.92 (0.77 to 1.07)	1.06 (0.81 to 1.31)	1.07 (0.51 to 1.64)
50-199	0.98 (0.97 to 1.00)	1.07 (1.03 to 1.12)	1.05 (0.99 to 1.11)	1.05 (0.94 to 1.16)
200-399	1.00 (0.99 to 1.02)	1.08 (1.05 to 1.11)	1.06 (1.01 to 1.11)	0.88 (0.77 to 0.99)
≥400	1.02 (1.00 to 1.04)	1.11 (1.09 to 1.14)	1.09 (1.04 to 1.14)	1.06 (0.97 to 1.16)

Abbreviations: NA, not applicable; OE, observed-to-expected.

^a^ Nonteaching indicates no residents training and the hospital does not belong to or partner with a university; residency, hospital has formal resident training programs but does not belong to a university; academic, hospital belongs to a university and trains residents.

^b^ Division indicates in or adjacent to area with at least 2.5 million people; metropolitan, area with at least 50 000 and less than 2.5 million people; micropolitan, area with at least 10 000 and less than 50 000 people; and rural, area with less than 10 000 people.

## Discussion

A wide range of factors contribute to the heightened risk of readmissions among adults with diabetes. In this large cohort of commercially insured adults and Medicare Advantage beneficiaries with diabetes across the United States, patient-level characteristics, clinical characteristics, patient economic status, factors surrounding their index hospitalization, and the characteristics of hospitals where they received care all affected the odds of unplanned all-cause 30-day readmission. Importantly, even after adjustment for these categories of readmission risk factors, black patients were significantly more likely to be readmitted than members of other racial/ethnic groups. This statistically significant increase in readmission risk could not be explained by other demographic factors, comorbidities, income, reason for index hospitalization, or place where they received care, although black patients did also have a higher prevalence of all these readmission risk factors than members of other racial/ethnic groups. Black patients with diabetes had a higher than expected readmission rate irrespective of where they received care, with the exception of very small hospitals (ie, <50 beds), where readmission rates were low for all patients. These findings in readmission rates and outcomes are concerning, particularly as they likely underestimate the true impact of racial/ethnic differences in the United States, as our study comprised privately insured individuals with access to care. (It is estimated that approximately 10% of black US residents are uninsured compared with 6% of white US residents.^29^)

Our study design did not allow us to examine the degree to which the increased risk of 30-day readmissions among black patients is distinct from their underlying heightened risk of any hospitalization. As such, black patients may face higher readmission risk because their risk of any hospitalization (index or recurrent) is higher. Nonetheless, examining 30-day unplanned readmissions specifically is important because these hospitalizations are potentially preventable in ways that index hospitalizations are not. Specifically, each hospitalization presents an opportunity for clinicians and health systems to identify individuals at high risk for rehospitalization and intervene by identifying potentially modifiable hospitalization and readmission risk factors, whether clinical, economic, or psychosocial.

Prior studies examining racial/ethnic disparities in hospital readmissions have focused almost exclusively on readmissions following acute myocardial infarction, heart failure, surgery, and pneumonia.^12,30,31,32,33,34,35^ To our knowledge, few have focused on patients with diabetes or assessed readmissions after other causes of hospitalizations, although those compose most inpatient care.^36^ The studies that did focus on these questions found higher hospitalization rates among black and Hispanic patients with diabetes compared with white patients.^6,37^ However, to our knowledge, none of these studies examined the association of race with readmissions separately from other important risk factors, such as age, comorbidity, economic factors, reasons for hospitalization, and characteristics of hospitals where patients receive care. Our study filled this important knowledge gap by demonstrating that while differences in demographic characteristics, clinical factors, economic status, and sites of care exist among people of different racial/ethnic groups and that these differences themselves are associated with heightened readmission risk, they do not explain the totality of excess readmission risk among black patients in the United States, which is not observed among other minority groups.

Site of care (eg, hospital characteristics) played an important role in readmission risk, particularly among black patients and, to a lesser degree, Hispanic patients. Nonprofit (compared with public and for-profit) hospitals, teaching hospitals (academic and, to a lesser degree, residency-only institutions), urban hospitals (located in division and metropolitan areas), and large hospitals (>400 beds) all had disproportionately higher rates of readmissions among black and Hispanic patients compared with white patients. Notably, rural hospitals had lower than expected readmission rates for white patients, but not for members of other racial/ethnic groups admitted there. These findings suggest underlying differences in how racial/ethnic minorities are treated in different settings, potentially because of different levels of resources, cultural knowledge and sensitivities, and community-based resources. Specifically, hospitals in deprived areas or areas without close community engagement in the health care system may lack appropriate resources to optimally manage the social and clinical complexities affecting minority patients with diabetes. Systemic attitudes and biases may also lead to inadequate support of patients following hospitalization, predisposing them to ultimate readmission. These factors need to be better understood, acknowledged, and ultimately addressed through policies and processes aimed at reducing disparities in health care delivery and outcomes.

Consistent with prior studies of patients with diabetes,^8,38,39^ younger patients had a higher risk of readmission compared with older patients once other risk factors for readmission, particularly comorbidities, were accounted for. This may reflect the unique challenges that younger people face when living with a chronic disease. This includes the demands of completing schooling and/or finding employment, building a family, facing uncertainty regarding finances and/or health insurance, and newly navigating independent life with chronic illness. Therefore, young adults living with chronic disease may benefit from additional resources and support, including clinical care processes better aligned with their daily lives as well as greater health care access and affordability. We also found that all diabetes-related and diabetes-unrelated comorbidities were associated with increased readmission risk. These findings were not surprising, considering extensive literature describing the effects of multimorbidity on readmission risk among patients without diabetes.^35,40,41,42^ However, the fact that black patients had a higher readmission risk after all of these factors were accounted for reinforces the existing differences in readmission rates and outcomes among black patients.

Black and Hispanic patients were more likely to be hospitalized specifically for diabetes-related causes, which may be preventable with high-quality ambulatory care.^36^ This is consistent with population-level data demonstrating worse diabetes care and health outcomes among racial/ethnic minorities.^2,3^ Our study built upon prior work that shows that these disparities correlate with, but are not entirely explained by, measurable social determinants of health.^5,6,9,10,11^ These findings further reinforce the importance of targeted efforts to improve diabetes management among black and Hispanic patients, who are at the highest risk for diabetes-related and all-cause hospitalizations and, as such, can inform allocation of local and national resources to patients with the highest risk and the hospitals where they seek care.

### Limitations

Our study has several limitations. First, this is a retrospective study that cannot establish a causal relationship between readmission risk and individual comorbidities, patient characteristics, and hospital features. However, it does identify and highlight important areas for clinical and policy interventions and improvement. Second, although the OptumLabsData Warehouse is among the largest administrative claims data assets, it is not entirely representative of the US population as a whole. In particular, patients without health insurance or those insured by Medicare, Medicaid, the Veterans Health Administration, and the Indian Health Service are not included. Many of these patients face additional barriers to health and health care, which may not be captured in our analyses. Third, potentially meaningful clinical and psychosocial factors cannot be captured using administrative data and therefore were not assessed; these include the availability of and ease of access to primary care physicians and specialists, detailed indicators of financial status, satisfaction with care, performance and functional status, health literacy, and social support.

## Conclusions

This study found that statistically significant and policy-relevant ethnic/racial differences exist in 30-day all-cause hospital readmissions, particularly among black adults with diabetes, and they did not improve over the period of the study. The reasons for these differences are multifactorial. Patients’ demographic, clinic, and economic factors as well as the characteristics of hospitals where they received care were all associated with increased readmission risk, disproportionately so among black and, to a lesser degree, Hispanic patients. These factors need to be acknowledged, investigated, and addressed to improve the equity and quality of diabetes care. Nevertheless, even after accounting for these measurable contributors to readmission risk, black patients were at an increased risk of hospital readmission, indicative of residual disparities that were not assessed. While this work does not provide solutions to the persistent disparities in readmission risk among black individuals with diabetes compared with white individuals with diabetes, it serves as a framework for future research and efforts aimed to reduce them.

## Notes

Supplement. **eTable. **Top Index Admission Diagnoses by Racial/Ethnic Group
