# Supplementary material for: Racial and Ethnic Differences in 30-Day Hospital Readmissions Among US Adults With Diabetes
Source: JAMA Netw Open. 2019 Oct 11;2(10):e1913249. doi: 10.1001/jamanetworkopen.2019.13249 (PMC6804020; doi:10.1001/jamanetworkopen.2019.13249)
Supplement: Supplement. — eTable. Top Index Admission Diagnoses by Racial/Ethnic Group [file jamanetwopen-2-e1913249-s001.pdf]

Supplementary Online Content

Rodriguez-Gutierrez R, Herrin J, Lipska KJ, Montori VM, Shah ND, McCoy RG. Racial and ethnic differences in 30-day hospital readmissions among US adults with diabetes. *JAMA Netw Open*. 2019;2(10):e1913249. doi:10.1001/jamanetworkopen.2019.13249

eTable. Top Index Admission Diagnoses by Racial/Ethnic Group

This supplementary material has been provided by the authors to give readers additional information about their work.

© 2019 Rodriguez-Gutierrez R et al. *JAMA Network Open.*

eTable. Top Index Admission Diagnoses by Racial/Ethnic Group

White patients	N (%)
Osteoarthritis	18039 (5.5)
Congestive heart failure	16926 (5.1)
Septicemia (except in labor)	14456 (4.4)
Diabetes mellitus with complications	13626 (4.1)
Pneumonia	13528 (4.1)
Coronary atherosclerosis	12254 (3.7)
Cardiac dysrhythmias	12088 (3.7)
Chronic obstructive pulmonary disease	11354 (3.4)
Acute myocardial infarction	9932 (3.0)
Skin and subcutaneous tissue infection	9192 (2.8)
**Black patients**	
Congestive heart failure	6109 (6.8)
Diabetes mellitus with complications	5236 (5.8)
Septicemia (except in labor)	3544 (3.9)
Acute and unspecified renal failure	3275 (3.6)
Osteoarthritis	3216 (3.6)
Pneumonia	3041 (3.4)
Chronic obstructive pulmonary disease	2854 (3.2)
Coronary atherosclerosis	2629 (2.9)
Cardiac dysrhythmias	2546 (2.8)
Acute cerebrovascular disease	2354 (2.6)
**Hispanic patients**	
Diabetes mellitus with complications	1952 (5.1)
Congestive heart failure	1856 (4.9)
Septicemia (except in labor)	1650 (4.3)
Coronary atherosclerosis	1429 (3.7)
Pneumonia	1425 (3.7)
Osteoarthritis	1399 (3.7)
Acute myocardial infarction	1091 (2.9)
Urinary tract infections	1075 (2.8)
Skin and subcutaneous tissue infection	1059 (2.8)
Nonspecific chest pain	995 (2.6)
**Asian patients**	
Septicemia (except in labor)	655 (6.6)
Coronary atherosclerosis	478 (4.8)
Congestive heart failure	470 (4.7)
Pneumonia	366 (3.7)
Diabetes mellitus with complications	358 (3.6)
Osteoarthritis	336 (3.4)
Cardiac dysrhythmias	305 (3.1)
Acute myocardial infarction	299 (3.0)
Acute cerebrovascular disease	275 (2.8)
Acute and unspecified renal failure	263 (2.6)

© 2019 Rodriguez-Gutierrez R et al. *JAMA Network Open.*
